# Detection of infectious bronchitis virus 793B, avian metapneumovirus, *Mycoplasma gallisepticum* and *Mycoplasma synoviae* in poultry in Ethiopia

**DOI:** 10.1007/s11250-016-1195-2

**Published:** 2016-12-06

**Authors:** S. Hutton, J. Bettridge, R. Christley, T. Habte, K. Ganapathy

**Affiliations:** 1 Institute of Infection and Global Health, School of Veterinary Science, University of Liverpool, Leahurst Campus, Neston, Cheshire CH64 7TE UK; 2 NIHR Health Protection Research Unit in Emerging and Zoonotic Infections, Liverpool, UK; 3 Ethiopian Institute of Agricultural Research, Debre Zeit, Ethiopia

**Keywords:** IBV, aMPV, Mg, Ms, Ethiopia, Serology, PCR

## Abstract

A survey was conducted into respiratory infectious diseases of poultry on a chicken breeder farm run by the Ethiopian Institute of Agricultural Research (EIAR), located in Debre Zeit, Ethiopia. Oropharyngeal swabs were collected from 117 randomly selected birds, and blood was taken from a subset of 73 of these birds. A combination of serological and molecular methods was used for detection of pathogens. For the first time in Ethiopia, we report the detection of variant infectious bronchitis virus (793B genotype), avian metapneumovirus subtype B and *Mycoplasma synoviae* in poultry. *Mycoplasma gallisepticum* was also found to be present; however, infectious laryngotracheitis virus was not detected by PCR. Newcastle disease virus (NDV) was not detected by PCR, but variable levels of anti-NDV HI antibody titres shows possible exposure to virulent strains or poor vaccine take, or both. For the burgeoning-intensive industry in Ethiopia, this study highlights several circulating infectious respiratory pathogens that can impact on poultry welfare and productivity.

## Introduction

Intensification of agriculture in Africa is accelerating, thanks to novel technologies, financial initiatives, changing social infrastructure and private sector engagement (Pretty et al. 2011), and policies promoting increases in consumption and productivity for a range of livestock species (Sumberg and Thompson 2013). Ethiopian entrepreneurs are setting up large, intensively managed flocks of exotic breeds, particularly in areas close to Addis Ababa (Mammo 2012). Government-owned poultry multiplication centres throughout the country (Pagani and Wossene 2008), non-governmental organisations and private individuals also distribute intensively reared chickens to smallholders (Pagani and Wossene 2008; Anon 2014). As a result, more urban and suburban households now keep flocks of 50 to 1000 birds under semi-intensive management (FAO 2008). The close links between intensive and smallholder farms facilitate disease spread, exacerbated by low biosecurity, and poor access to veterinary inputs and expertise among smaller producers (Sambo et al. 2014).

Respiratory infections have major impacts on poultry welfare and productivity, yet are largely unstudied in most African countries (Owoade et al. 2006), where attention has focussed on the notifiable avian influenza (AI) and Newcastle disease (ND) viruses. Other common respiratory pathogens worldwide include infectious bronchitis virus (IBV), infectious laryngotracheitis virus (ILTV) and avian metapneumovirus (aMPV) (Cavanagh and Gelb Jr. 2008; Gough and Jones 2008; Guy and Garcia 2008). These cause direct losses also interact with bacteria such as *Mycoplasma gallisepticum* (Mg) and *Mycoplasma synoviae* (Ms), which exacerbate respiratory diseases. Serology shows ND is widespread throughout Ethiopia (Tadesse et al. 2005; Zeleke et al. 2005; Regasa et al. 2007; Mazengia et al. 2010; Chaka et al. 2012), and virulent and lentogenic strains have been isolated (Chaka et al. 2013; Fentie et al. 2014). Vaccines, produced locally, are used in commercial farms, multiplication centres (Dessie and Jobre 2004) and more recently in field trials for village birds (Nega et al. 2012). Low pathogenicity AI was last reported in 2006 and high pathogenicity AI has never been identified in Ethiopia. ILT and IBV (serotype not stated) were last reported in 2000 and 2001, respectively, but no information is available on aMPV (O.I.E. 2015), which is under-reported in many parts of the world due to a lack of diagnostic facilities (Gough and Jones 2008). To our knowledge, the scientific literature contains no reports of these last three in Ethiopia; therefore, circulating genotypes, important for identification of appropriate vaccines, are entirely unknown. Mg has been identified in sick birds from commercial farms and in village chickens from live markets both serologically (Alamargot et al. 1985) and through culture and PCR (Bekele 2015). Ms appears to be previously unreported in Ethiopia.

The poultry farm at the Ethiopian Institute of Agricultural Research (EIAR), Debre Zeit, represents an important centre for research, farmer training in poultry production and distribution of birds among smallholders. As such, it has vital links with farmers, especially intensive and semi-intensive producers. In 2012, respiratory disease was a major cause of morbidity and mortality; therefore, this study screened the EIAR flocks for IBV, aMPV, Newcastle disease virus (NDV), ILTV, Mg and Ms.

## Materials and methods

### Flock and samples

Sampling was conducted over a 2-week period in May 2012. At the time of study, the farm consisted of 11 sheds, nine of which were populated. Three sheds which housed grandparent birds were situated separately to the main body of the poultry production premises. The main premises consisted of a hatching house; incubator and brooder sheds (both disused); one large new breeder shed; two multi-age multi-breed sheds containing parent birds and multiplier flocks and two sheds containing indigenous chicken ecotypes. These last two were not sampled due to local concern over biosecurity, as any losses incurred in this flock would interrupt an experimental breeding programme. All birds on the farm were vaccinated against NDV, but unfortunately records of vaccination schedules for the birds sampled were not available.

Groups were defined as birds of the same age and breed. These were mostly kept in the same shed, with the exception of some groups, where birds were split between the parent and multiplier sheds. There were seven sheds sampled in total, and the number of groups varied per shed. All groups were of mixed sex and, depending on the group size, were housed in up to eight pens within the same shed (Table 1.) Where groups were split between pens, at least one bird was selected from each pen. Birds were selected from pens by convenience sampling; avoiding birds by the pen doors and using the most competent keepers for catching to reduce selection of slow, weak birds and thus selection bias. Selected birds were first swabbed oropharyngeally and then blood sampled (1 ml via brachial vein). Blood samples were collected into a plain syringe and allowed to clot at room temperature before the serum was removed and stored at −20 °C. The majority of grandparent and breeder birds were both swabbed and blood sampled, whereas only around half of the parent birds were bled. These were kept in more, but smaller groups, thus three to five sera per pen were collected and two to four additional birds per pen were swabbed. Chicks were not blood sampled, due to possible adverse effects of the bleeding procedure and interference of maternally derived antibodies with serology results.

**Table 1:** Breeder flocks examined for serological evidence of exposure to NDV, IBV or aMPV by ELISA and the presence of IBV, aMPV, Mg and Ms by PCR

House	Group	Breed	Age (months)	Number of birds examined for NDV, IBV and aMPV ELISA antibodies	NDV vaccination index	PCR detection in pooled OP swabs collected from *n* of birds				
						*n*	IBV	aMPV	Mg	Ms
Grandparent 1	1^a^	Dominant CZ Rhode Island Red	22.5	10	133.2	10	−	−	−	+
Grandparent 2	2	Dominant CZ	22.5	10	278.1	15	+	−	−	−
Grandparent	3^b^	Hubbard JV	9	10	43.5	10	−	−	−	+
Breeder	4^c^	Hubbard JV	9	20	40.1	20	−	−	−	+
Parent (multi-age in single house)	5	Hybrid	7	3	12.3	5	−	−	+	+
	6	Kokok	23	3	15.1	7	−	−	−	+
	7	Hybrid	9	3	128.1	5	−	−	−	+
	8	Fayoumi	7	3	34.9	6	−	−	+	+
	9	Kokok	31	3	55.0	5	−	−	−	+
	10	Fayoumi	31	5	5.8	8	−	−	−	+
	11	Dominant CZ Rhode Island Red	28	3	64.5	6	−	−	−	+
Chick	12	Hybrid	0.5	Not done	Not done	20	−	−	−	+

^a^One dead bird was necropsied; no IBV, aMPV, Mg or Ms was detected by PCR

^b^One dead bird was necropsied; no IBV, aMPV or Mg was detected, but Ms was detected in the trachea and turbinate by PCR

^c^One dead bird was necropsied; no Mg or Ms was detected, but IBV 793B and aMPV subtype B were detected in the turbinate by PCR

### Serum antibody detection

ELISA kits (BioChek, Reeuwijk, The Netherlands) were used to detect antibodies for NDV, IBV and aMPV in 73 sera. Tests were carried out in a laboratory within the EIAR research facility in Debre Zeit. Tests were performed according to the manufacturer’s instructions, but with the following modifications: all incubation times for NDV and IBV plates were reduced by 5 min, and a 10-min reduction was applied to aMPV plate incubation periods. This was to compensate for the higher-than-recommended room temperature of the laboratory (30 °C instead of between 17 and 24 °C). Samples were tested in triplicate, and individual antibody titres were calculated according to the manufacturer’s instructions. For the NDV antibody results, as birds were vaccinated against this pathogen, the vaccination index [VI = (mean titre)^2^/(St Dev × 100)] was calculated for each group according to van Leerdam and Bosman (2006), which relates the mean group titre to the coefficient of variation, and so provides a simple evaluation of the effectiveness of a vaccination.

### Antigen detection

Oropharyngeal swabs were pooled; two to three swabs from birds within the same group were placed in a sterile bijou containing 2 ml of sterile water, vortexed for 3 s and 80 μl of supernatant was transferred onto an FTA card. In addition, three birds isolated in hospital pens within the grandparent and breeder sheds were sacrificed by cervical dislocation and post mortem examination was carried out. Tissues of the trachea (*n* = 3), lung (*n* = 3), oviduct (*n* = 3), kidney (*n* = 3) and turbinate (*n* = 2) were collected, ground until smooth with a pestle, sterile sand and 3 ml of sterile distilled water. After two freeze-thaw cycles and centrifugation at 2000*g* for 5 min, 80 μl of each supernatant was applied to an FTA card. FTA cards, stored at room temperature, were transported under license to the Poultry Respiratory Diseases Group, University of Liverpool, UK, for further processing.

Forty-one pooled oropharyngeal and 14 processed tissue samples on FTA cards were processed, where each circle of the FTA card containing a sample was removed using sterile scissors and forceps and placed in a bijou containing 800–1000 μl of TE buffer (10 mM Tris–HCl, 0.1 mM EDTA, pH 8.0), vortexed and incubated at room temperature for 10 min (Abdelwhab et al. 2011). The supernatant was then used to extract viral RNA for detection of NDV, IBV and aMPV. For IBV, NDV and aMPV detection, RNA extraction was performed on TE buffer using the QIAamp Viral RNA Mini Kit (Qiagen Ltd., Hilden, Germany), and for detection of ILTV and both Mg and Ms, DNA was extracted from TE buffer using the QIAamp DNA Mini Kit (Qiagen Ltd., Germany). All extractions were according to the manufacturer’s instructions and stored at −20 °C until required.

The extracted RNA or DNA were tested for PCR using s for NDV (Aldous et al. 2003), ILTV (Diallo et al. 2010), IBV and aMPV (Cavanagh et al. 1999; Worthington et al. 2008; Al-Shekaili et al. 2015a; Ganapathy et al. 2015). A commercial PCR kit (Adiavet-myco-AV PCR, Adiagen, St Brieuc, France) was used to detect the presence of Mg or Ms DNAs. Positive IBV PCR reactions were sequenced, analysed and compared against reference strains as described before (Al-Shekaili et al. 2015a; Ganapathy et al. 2015).

## Results

### aMPV

**Fig. 1:**
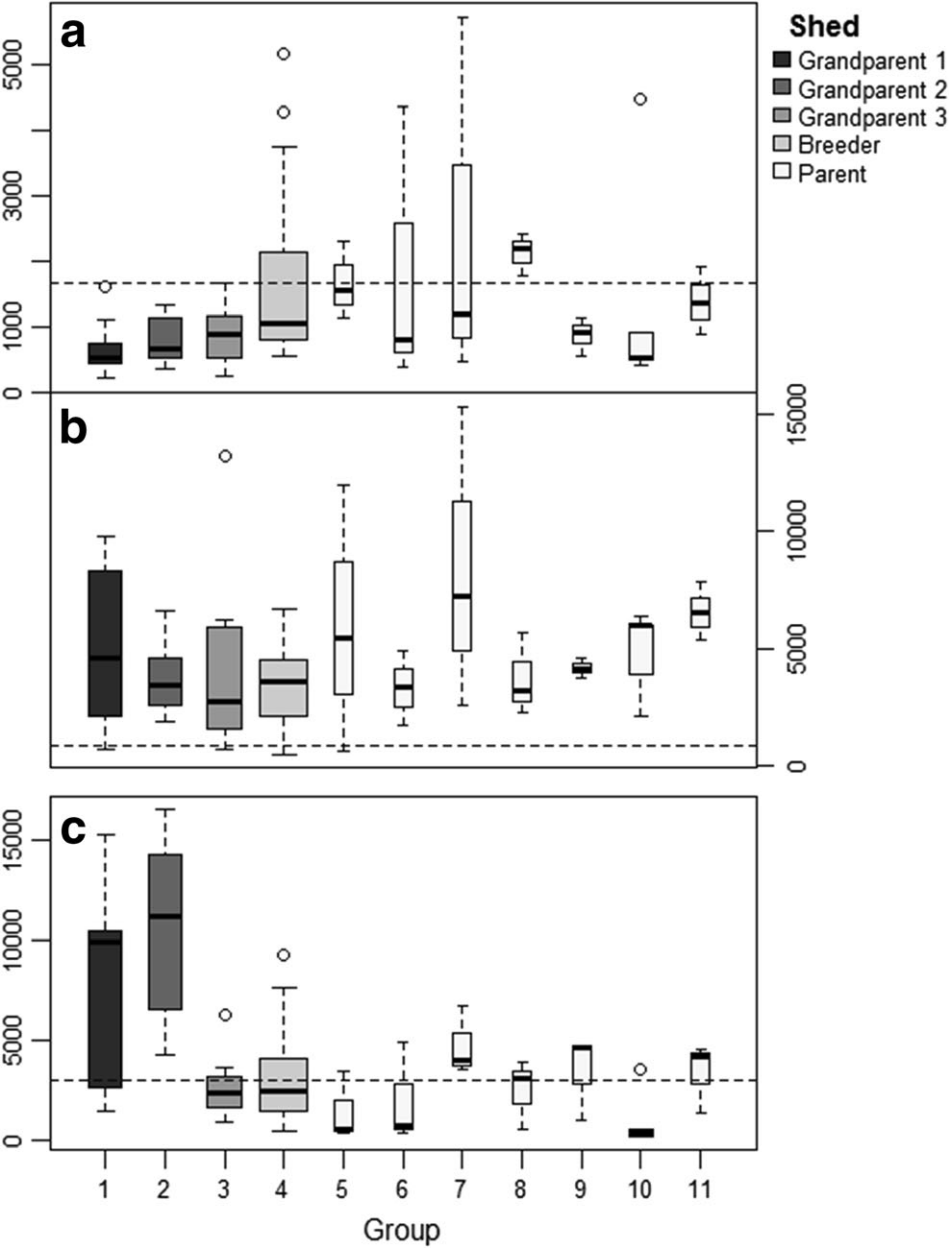
Antibody titres for 73 birds against **a** avian metapneumovirus, **b** infectious bronchitis virus and **c** Newcastle disease virus. *Widths of the bars* represent the number of samples tested for each group. *Dotted horizontal lines* show positive cut-off values

Serum samples were considered to be positive for aMPV if titres ≥1656, as per the manufacturer’s guidelines. Sixteen (21.9%) samples tested positive (Fig. 1a). All the OP swabs were negative for aMPV by RT-PCR. A turbinate from a necropsied bird from group 3 was positive for aMPV subtype B.

### IBV

Using the manufacturer’s cut-off of 834, 69 (94.5%) birds were serologically positive for IBV (Fig. 1b). By RT-PCR, only one (group 2) of the pooled OP swab was positive for IBV. Positive PCR reaction was also detected in the same turbinate sample from group 3, which was also positive for aMPV (above). Sequencing revealed these IBVs belong to 793B with 92–95% relatedness to the French isolate FR-94047-94 (GenBank accession number: AJ618987).

### NDV

ELISA results demonstrated considerable variation in the antibody titres, both within and between the groups (Fig. 1c). Three of the groups within the parent shed (groups 5, 6 and 10) had a VI below the lower limit of 20 suggested by the manufacturer for birds vaccinated with live vaccine (Table 1). One of the grandparent groups (group 2) was outside the upper limit of the normal range (250) with a VI of 278. However, this was below the cut-off value of 300, values above which might suggest field challenge. No NDV was identified in the OP or tissue samples by RT-PCR.

### ILTV

Serological testing for ILTV was not performed. No ILTV detected in any of the samples when examined by PCR.

### Mycoplasmas

No Mg or Ms serology was carried out. PCR examination of oropharyngeal swabs identified Mg in the two groups of birds in the parent shed (groups 5 and 8). Ms was identified in 11 of the 12 groups; only one group of grandparent stock (group 2) was negative. Ms was also identified in the trachea and turbinate samples of one of the sacrificed hospitalised birds from one of the grandparent sheds.

## Discussion

To our knowledge, this is the first publication to report IBV and aMPV in Ethiopia, and the first time that aMPV and Ms have been identified in the country. Sequencing data of IBV isolates identified 793B genotype with a 92–95% similarity to a French isolate FR-94047-94, which is closely related to virulent 4/91 (Cavanagh et al. 2005). IBV vaccine is not used on the farm; therefore, this is presumed to be a field infection. The strain 793B is one of the most common strains and has previously been isolated from West African countries (Ducatez et al. 2009) and Tunisia (Bourogâa et al. 2009). The aMPV subtype B is a very common strain in other parts of Africa and was also unlikely to have been vaccinated in origin, as aMPV vaccines are not in use on this farm, and to the best of our knowledge, not currently available in Ethiopia. Knowledge of the strains present can help inform vaccine selection in the event that vaccination programmes for respiratory pathogens are implemented in the future.

Both Mg and Ms were identified on this farm, with Ms being found in almost all sampled groups. Both these respiratory pathogens transmit readily in birds within the same airspace, with up to 100% of birds in a flock becoming infected (Kleven and Ferguson-Noel 2008). Human traffic is also an important contributor to spread between sheds (Mohammed et al. 1987). Other risk factors for *Mycoplasmas* include overcrowding, poor ventilation and hygiene, introduction of new birds and multiple age flocks. High standards of hygiene and biosecurity are essential for controlling the spread of these pathogens. In addition, as vertical transmission can also occur (Michiels et al. 2016), maintaining breeding stocks free of infection is desirable. However, in situations where multiple ages and types of poultry are in close proximity to each other and to backyard flocks, this may be difficult or impossible, and vaccinations or antimicrobials may be required to reduce transmission (Kleven and Ferguson-Noel 2008; Ley 2008). The EIAR farm is primarily a research station and trials different breeds imported from different parts of Ethiopia and abroad. Therefore, it has to continually receive and maintain several small groups of poultry of different ages, and even with quarantine precautions, it is unlikely that eradication will be feasible, as an all-in all-out system will never be practical with the current housing facilities.

The extreme variability and low VI of at least three groups of birds sampled would suggest that improvements could be made in the effectiveness of the NDV vaccine administration or problems with the vaccines themselves. Although part of this variation will be due to the mix of ages and types of birds kept, route and frequency of administration, it may be desirable for the farm to investigate this finding further. Whilst the presence of NDV was not detected (by PCR) on the farm, our findings would suggest that a proportion of birds may be vulnerable to virulent NDV challenges. The range and magnitude of the serological results also provided evidence to suggest repeat exposure of the birds to IBV and aMPV, supported by antigenic identification of both pathogens, suggesting circulation within the flocks. Again, increased biosecurity measures may be of assistance in reducing transmission, but may be insufficient alone, due to the multiple ages of flocks making complete disinfection of premises impossible. Other management practices, such as ensuring adequate ventilation and reducing overcrowding, and the use of vaccination may be necessary to reduce production losses (Cavanagh and Gelb Jr. 2008).

Further studies are needed to establish the presence of these pathogens in other flocks in the local area and further across the region. A recent review of IBV in Africa (Khataby et al. 2016) contained no papers from East Africa, although numerous strain variants, including some unique to particular countries, have been reported in North and West Africa and southern parts of the continent. Reports from other countries worldwide, such as Bangladesh, Mexico, Oman and Algeria, where the poultry sectors are transforming, all indicate that these respiratory pathogens are a common and widespread source of concern to the developing industries (Islam et al. 2014; Rivera-Benitez et al. 2014; Al-Shekaili et al. 2015b; Sid et al. 2015). Ethiopia and other countries in East Africa are likely to face the same problems as intensification of poultry farming continues.

Future sampling should include backyard stock, as birds are frequently distributed from this, and other poultry centres, to smallholders, where they mix with the indigenous populations. Poultry traders are also known to sell chickens intended for retail to smallholder producers for breeding, and disease outbreaks in villages are often suspected to be caused by poultry introduced from markets to a household in the village (Chaka et al. 2011). Whilst trading live birds through markets is a known risk for introducing NDV, it is also highly likely to disseminate other pathogens, which are largely unstudied in village poultry. For the burgeoning-intensive industry, initiating control programmes involving higher biosecurity and vaccination for these important respiratory pathogens may be an essential component to boost welfare and productivity, and trialling such programmes at this research centre would be an ideal way to promote and disseminate such information among local producers through the existing training programmes available at the centre.
